# Exploring pattern recognition: what is the relationship between the recognition of words, faces and other objects?

**DOI:** 10.1007/s10339-022-01111-3

**Published:** 2022-11-14

**Authors:** F. A. Maratos, K. Chu, S. Lipka, E. J. N. Stupple, F. Parente

**Affiliations:** 1 School of Psychology, College of Health, Psychology and Social Care, University of Derby, Derby, UK; 2 Hong Kong Polytechnic University, Hong Kong, China

## Abstract

Debate surrounds processes of visual recognition, with no consensus as to whether recognition of distinct object categories (faces, bodies, cars, and words) is domain specific or subserved by domain-general visual recognition mechanisms. Here, we investigated correlations between the performance of 74 participants on recognition tasks for words, faces and other object categories. Participants completed a counter-balanced test battery of the Cambridge Face, Car and Body Parts Memory tests, as well as a standard four category lexical decision task, with response time and recognition accuracy as dependent variables. Results revealed significant correlations across domains for both recognition accuracy and response time, providing some support for domain-general pattern recognition. Further exploration of the data using principal component analysis (PCA) revealed a two-component model for both the response time and accuracy data. However, how the various word and object recognition tasks fitted these components varied considerably but did hint at familiarity/expertise as a common factor. In sum, we argue a complex relationship exists between domain-specific processing and domain-general processing, but that this is shaped by expertise. To further our understanding of pattern recognition, research investigating the recognition of words, faces and other objects in dyslexic individuals is recommended, as is research exploiting neuroimaging methodologies, with excellent temporal resolution, to chart the temporal specifics of different forms of visual pattern recognition.

## Introduction

Humans have a marked reliance on vision and visual experiences to interact with, understand, and navigate the world around them (San Roque et al., 2015). As such a plethora of research has focused on human visual experience, from the neurobiology of low-level perception to higher-level object recognition and categorisation (for a review see Griffin & Motta-Mena, 2019). This has led to three object recognition areas of study: (i) *functional* *specificity*, (ii) *anatomical* *stereotypy*, and (iii) *innateness*, mapping onto: (i) Are recognition mechanisms different for different object domains? (ii) If so, are these different mechanisms subserved by different brain regions that show anatomical consistency between individuals? (iii) Do specificity and stereotypy develop under genetic control or environmental influence?

**Fig. 1:**
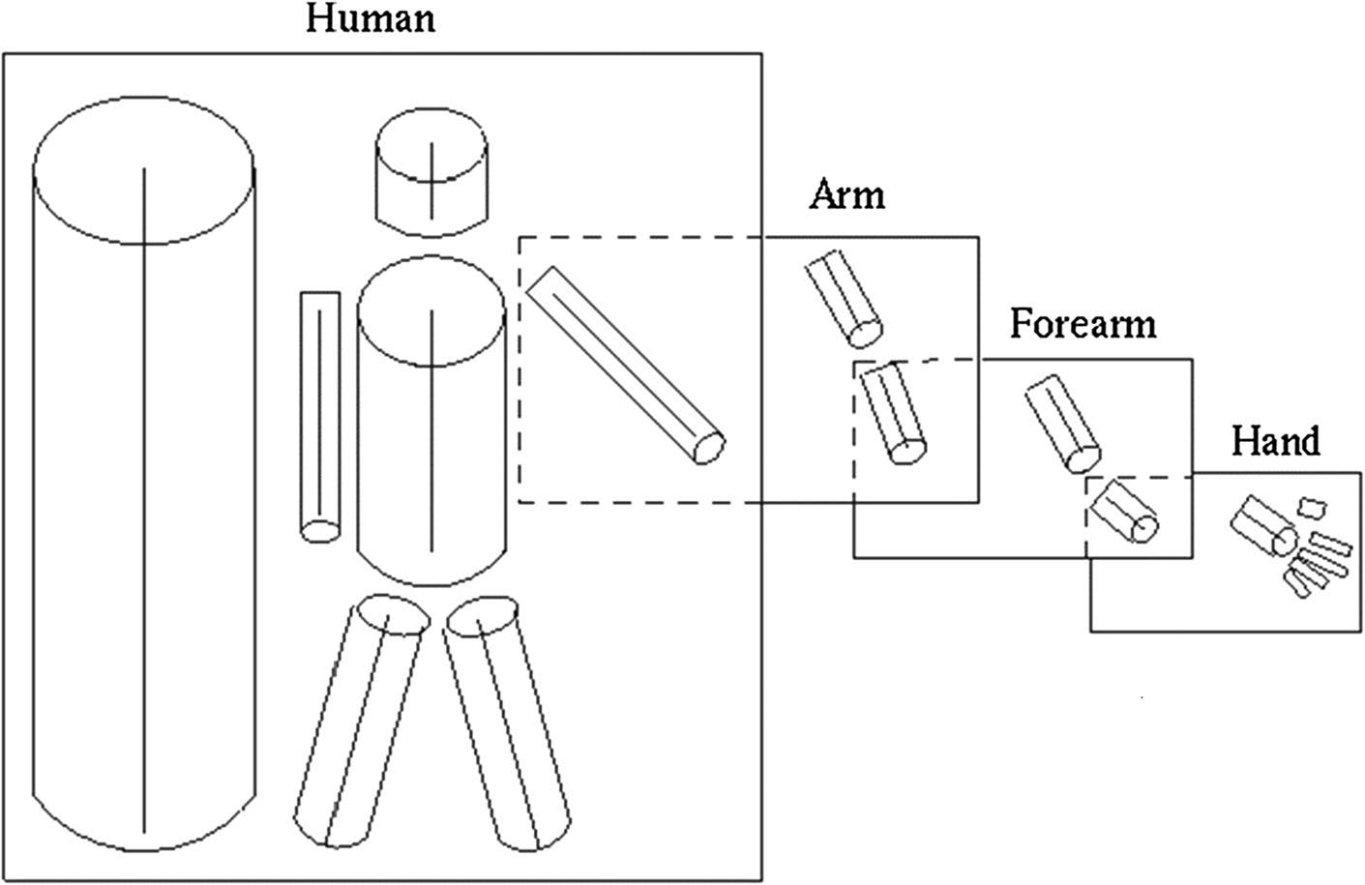
Example of how the visual recognition of complex objects (e.g., a human body) could be performed through decomposition into more basic visual primitives (i.e., volumes and shapes), a mechanism that could be domain-general. Figure reproduced with permission from Marr (1982)

Whilst the idea of functionally and anatomically distinct brain regions can be traced back to nineteenth-century neuroanatomical patient investigations (e.g., Broca 1861; see Dronkers et al., 2007), with respect to models of visual object recognition, Marr (1982; see also Biederman 1986) posited that visual input can be used to construct representations that decompose objects into visual primitives (see Fig. 1). This would be achieved through *general-purpose* *mechanisms* applicable to any object in the visual field regardless of category.

More recently, neuroimaging has provided researchers with a means to directly investigate questions of functional specificity as well as anatomical consistency. For example, an area in the occipital region, the lateral occipital complex (*LOC*), has been identified to preferentially respond to images of intact objects compared to scrambled images (of the same objects), demonstrating effects of object familiarity (Malach et al., 1995; Margalit et al., 2016), and category (Eger et al., 2008). Similarly, Sergent et al., (1992) identified an area of the fusiform gyrus (the ‘fusiform face area’; *FFA*) to demonstrate selective responses to faces as compared to non-face objects. Kanwisher et al., (1997) further observed that this area showed stronger responses in the right hemisphere and argued, consistent with functional specificity, that it forms part of a larger network of face-processing regions (see also Gauthier et al., 2000a, b; Rajimehr et al., 2009). More recently, Almeida and colleagues (2020) described a case of a patient with a left splenium lesion resulting in distorted processing specific to one side of human faces and not generalising to other classes of objects. Taken together, such studies support *functional* *specificity* for the identification of different object categories.

Correspondingly, Cohen et al., (2000) identified a region of the left fusiform gyrus that showed selectivity for written words (Bolger et al., 2005; Cohen and Dehaene 2004; Dehaene and Cohen 2011; Dien 2009)—the ‘visual word form area’ (*VWFA*). Further studies have since identified additional occipitotemporal regions as having functional specialisation for: landmarks (e.g., Epstein et al., 1999), visual scenes (e.g., Kamps et al., 2016), and numbers (e.g., Hannagan et al., 2015). In sum, some brain-based evidence points to innately programmed differential neural circuitry to process biologically important stimuli, arguably given their importance to survival and bonding (e.g., in the case of faces see: McKone et al., 2012; Owen and Maratos 2016).

This stated, further researchers have challenged notions of selectivity, consistency, and innateness, putting forward arguments for functional and anatomical overlap, as well as experience in shaping neural organisation (e.g., Aguirre et al., 1998) or mechanisms (e.g., Beauchamp et al., 2003; Chao et al., 2002; Downing et al., 2006).

To expand, whilst the FFA is hailed as a clear example of functional and anatomical specialisation, efforts to trace its evolution by identifying homologous areas in social non-human primates have yielded mixed results (Parr 2011; Rossion and Taubert 2019). Moreover, several studies have shown that areas in the face network (including the FFA) respond not only to faces, but other round objects (e.g., clocks or balls), potentially suggesting the existence of a common object recognition system based on primitive visual properties (Srihasam et al., 2014; Tsao et al., Livingstone, 2006; Yue et al., 2014) akin to a visual primitive argument (Marr 1982). Studies in macaque (Livingstone et al., 2017) and human infants (Deen et al., 2017) have further found that face selectivity fully develops over several months after birth, with face expertise actually developing during early adolescence in humans (Hills and Lewis 2018). Moreover, face-stimulus deprivation in macaques prevents the formation of a face-processing area (which is then repurposed in a persistent fashion for general object recognition; Arcaro et al., 2017). These findings argue against *functional* *specificity*; rather they are consistent with the expertise hypothesis put forward by Gauthier and colleagues (Gauthier et al., 1999).

In the expertise hypothesis, the FFA is part of a domain-general object recognition system, which is recruited for within-category object discriminations in domains of expertise. Here, face specialisation of the FFA gradually appears as infants/children develop more expertise in face processing and acquire familiarity with more and more individual faces to discriminate between. Consistent with an expertise hypothesis, further studies have shown that ‘novel’ object recognition correlations with face recognition performance depend upon a participant’s expertise with the ‘novel’ object category (e.g., Bilalić 2016; Bilalić et al., 2016; Gauthier et al., 2014; Martens et al., 2018; see also Wang et al., 2016 for a modelling study using a biologically plausible neural network). In sum, these studies point to a shared object recognition mechanism guided by low-level visual properties, with the FFA serving as a *domain-general* discrimination system.

Much like the FFA, the domain-specificity and purpose of the VWFA has been debated, especially as the invention of written language is too recent in evolutionary terms for a dedicated ‘written word recognition’ region to have been innately selected for. Instead, a recycling hypothesis has been proposed (Dehaene and Cohen 2007). Here, it is argued that cultural inventions such as reading take over evolutionarily older circuits that then become repurposed. For example, whilst the VWFA supports reading following literacy acquisition, it might also maintain other functions, such as the processing of groupings of visual stimuli characterised by high spatial frequencies and high complexity (Vogel et al., 2014; see also Price and Devlin 2003). This would include, but not be limited to, groups of letter symbols and faces (Keil et al., 2008).

The possibility that the VWFA might be involved in the processing of different classes of visual objects raises the possibility of associations between reading and object recognition more generally. Consistent with this, Dundas et al., (2013) presented evidence that the emergence of face lateralisation is correlated with reading competence. Accordingly, studies have begun to explore the linkage/overlap between face and language processing more generally (e.g., Dundas et al., 2014; Robinson et al., 2017), as well as across patient populations (e.g., Asperud et al., 2019; Roberts et al., 2015). Asperud et al., (2019), for instance, studied seven right-handed individuals with unilateral posterior cortex focal lesions and found evidence to support a bilaterally distributed network for the processing of words and faces. Importantly, however, they found that whilst in all cases word recognition deficits were paired with face deficits, the reverse was not true. Robinson et al., (2017) further observed face processing to interfere with word processing (but not vice-versa), suggesting a shared overlapping distributed network, especially for faces. Taken together, such findings suggest that object recognition relies on a broad network of functionally and anatomically overlapping brain regions, with many complex interactions between them.

This research fits with the many-to-many theory of object recognition (MTM; Behrmann and Plaut 2013). This theory proposes that, while there may be brain regions ideally suited to the processing of certain properties of visual stimuli (see also Arcaro et al., 2019; Jang et al., 2019), these regions represent multiple object classes that share similar perceptual features. However, the MTM theory cannot explain dissociations in recognition abilities between different object domains. For example, cases of specific visual agnosias are a common finding (Behrmann et al., 1994; Farah 1992; Moscovitch et al., 1997; Starrfelt et al., 2018; Susilo et al., 2015).

In sum, the literature surrounding pattern recognition, and various elements of such (e.g., words vs. face recognition) is complex and often contradictory. As such, it is important to investigate and/or clarify whether performance across different forms of visual pattern recognition is correlated at the behavioural level, using an appropriately powered sample and a variety of object classes. Thus, in the present study, we explored the relationship, if any, between word, faces and other object category recognition in the general population. We used the Cambridge Face, Car and Body Memory Tests (Dennett et al., 2012; Duchaine and Nakayama 2006) as well as a standard lexical decision task (as our measure of visual word recognition; see Harley 2014). In lexical decision tasks, participants need to decide if visually-presented letter strings are known (English) words or made-up letter strings (i.e., non-words).

We hypothesised that if the recognition of words, faces, and other object categories (i.e., bodies and cars) share common neural underpinnings, then performance across tasks should be correlated, especially for the more familiar tasks (e.g., face and known-word recognition). However, if these different forms of pattern recognition are distinct dissociable ability domains, then performance across tasks would not be correlated. Following on from an initial round of correlations, we then performed a principal component analysis (PCA). PCA is a multivariate analytical method that summarises the correlations between observed variables as a smaller set of “components” allowing commonalities between them, where observed, to be demonstrated. In our case, the goal was to identify commonalities—if any—in performance across the different tasks and/or task components.

## Method

### Design

We employed a correlational design. Sample size was calculated based on Pinel et al., (2015) and their non-word reading/FFA correlations. To obtain an expected correlation co-efficient of 0.32 between our key variables of (non-word) reading and face recognition with acceptable power (i.e., 0.8; with alpha set at 0.05), the calculated sample size required was 74.

### Participants

Participants were recruited using opportunity sampling. Inclusion criteria included being a native English speaker and of age 18 or over. Consequently, 74 native English speakers were recruited and completed the study in full. Eighteen were male (24%), and all participants were either university students (undergraduate/ postgraduate) or staff (both academic and non-academic) from the University of Derby, United Kingdom. Participants ranged in age from 18.5 to 72.5 years, with an average age of 36.96 years. All participants gave informed written consent to participate in the study and received course credits (where applicable) or book tokens for their participation. The study received University of Derby Human Sciences Research Ethics Committee approval.

### Materials

#### Visual and lexical recognition tasks

##### *Cambridge face memory test (CFMT),* Duchaine & Nakayama (2006)

The CFMT measures participants’ face recognition ability and has high internal reliability (Bowels et al., 2009). Items (and procedure) are the same as described in Duchaine and Nakayama (2006). Items consist of black and white photographs of neutral male faces taken in various poses (frontal viewing profile; 1/3 left profile; 1/3 right profile) and lighting conditions. *Target* *faces* consist of six male faces, with 12 images of each. *Distractor* *faces* consist of 46 different male images of the same age range, and taken in the same poses and lighting conditions as the target faces. All test items consist of a target face being presented alongside two distractor faces (randomly ordered), and below each a number (1, 2, 3) is presented. Scores can range from 0 to 72, with Duchaine and Nakayama (2006) reporting an average score of 57.9 in a sample of 50 University-aged students.

##### *Cambridge car memory test (CCMT),* Dennett et al., (2012)

The CCMT measures participants’ ability in car recognition with high internal reliability (Dennett et al., 2012). The format and stages of the CCMT are the same as the CFMT described above, except that photographic stimuli are cars rather than faces. Whilst various types of cars are used for the picture stimuli (e.g., sedans, sports cars, and wagons), they are presented in grayscale, with no identifying badges, logos and insignia. Scores can range from 0 to 72, with Dennett et al., reporting an average of 53.18 in a sample of 153 University-aged students.

##### *Cambridge body memory test (CBMT)*, Duchaine & Nakayama (unpublished)

The CBMT measures participants’ ability in body recognition and has the same format and stages as the CFMT and CCMT, with the exception that photographic stimuli are male bodies. Bodies are of various shapes and height, with only torso and limbs shown in grayscale. Scores can range from 0 to 72. To the authors’ knowledge, no descriptives are available for this task.

##### *Lexical decision task (LDT)*

The lexical decision task measures a participant’s visual word recognition ability and is the most common behavioural method for investigating visual word recognition (Norris, 2013). Stimuli consisted of four trial types comprising two types of words and two types of made-up non-words. These were: (1) regularly spelt words (e.g., peach), which follow regular grapheme-to-phoneme correspondence rules; (2) irregularly spelt words (e.g., bough), which do not follow regular grapheme-to-phoneme correspondence rules and require visual memory of the correct orthography of the whole word (visual route); (3) pseudo-homophone non-words (e.g., floot), which are unfamiliar strings of letters that sound like real English words if grapheme-to-phoneme correspondence rules are applied (in this case: flute), and (4) visually-confusable non-words (e.g., weeb; whem). This final category of non-words are unfamiliar strings of letters which do not sound like real English words but have high visual similarity to real words as they were created by either reversing letters that people with dyslexia often confuse (e.g., in weeb, the letter ‘*b*’ can be easily confused with ‘*d*’) or substituting letters with a visually similar letter (e.g., in whem, the letter ‘m’ has a high visual similarity with ‘n’). Importantly, all non-words were created such that they were pronounceable and orthographically legal, in line with standard practice (Gabay et al., 2017).

In the current task, a total of 92 stimuli were used: 80 trial stimuli (20 per trial type), 8 practice stimuli and 4 warm-up stimuli. Number of letters was identical across stimulus type (average 4.5, range 36), and stimuli were matched on lexical frequency using the Elexicon megastudy norms (Balota et al., 2007), with an average of 5665.85 in the Hyperspace Analogue to Language (HAL) frequency norms and 7.72 in the log-transformed HAL frequency norms. Accuracy scores could range from: 0–20 per trial type; 0–40 per word type; 0–80 for overall task performance. Additionally, *correct* *answer* reaction time data were recorded.

##### Questionnaire measures

##### *Waterloo handedness questionnaire-revised (WHQ-R)*, Elias et al. (1998)

This questionnaire contains 36 items to measure participants’ hand preference when executing various skilled (e.g., manipulation of objects) and less skilled (e.g., pick up small objects) activities. The test has high reliability (*r* = 0.88) (Steenhuis et al., 1990). Participants rate their hand preference according to a 5-point scale (Always left/ Usually left/ Equal/ Usually right/ Always right). Responses are scored by assigning values from  −  2 to 2 (Elias et al., 1998). Thus, total scores can range from − 72 (strongly left-handed) to 72 (strongly right-handed).

### Procedure

Participants were asked to sit at a distance of approximately 49.5 cm away from the computer screen with task presentation counter-balanced. That is, the face, car and body visual recognition tasks were counter-balanced, and either preceded or followed the LDT in 50% of cases, respectively. The Cambridge tasks were programmed using Java (https://www.java.com/), whereas the LDT was programmed using Inquisit (https://www.millisecond.com/).

In the CFMT, CCMT and CBMT participants were instructed to memorize the faces, cars or bodies that would follow. These tasks involved four phases, a Practice-, Introduction-, Novel- and Novel with Gaussian noise-phase, respectively. The latter three phases comprised 72 trials and the practice phase three trials. In a practice trial, three study images of a target stimulus (a frontal viewing profile, a 1/3 left profile and a 1/3 right profile) were presented consecutively for three seconds each. Participants were then asked to discriminate the target stimulus (shown in one of the prior viewing profiles) from two distractor stimuli by pressing keyboard number ‘1’, ‘2’, or ‘3’ that corresponded to the number below each stimulus image. Participants needed to score 100% (i.e., 3 out of 3 trials correct) before they could progress to the actual test. In the actual task, the ‘Introduction’ phase (comprising 18 trials with 6 targets × 3 presentations) had the same format as the practice trials. That is, participants viewed three images of a face, car or body part in three different orientations and then discriminated this target from two distractors (of the same category) with the same lighting and viewing profile. Following on from this, in the ‘Novel’ phase (comprising 30 trials with 6 targets × 5 presentations), six targets were presented simultaneously in a single review image from a frontal profile for twenty seconds. After which a participant was presented with three stimuli and asked to discriminate the target (i.e., one of the six prior stimuli) from two distractor stimuli. Importantly in this ‘novel’ version, distractor stimuli were novel images of the same category, but with different lighting and/or viewing profiles. Finally, in the ‘Novel with Gaussian noise’ phase (24 trials: 6 target faces × 4 presentations), trial events were the same as in the novel condition, but with the exception that all images were covered with Gaussian noise during the discrimination process to prevent ceiling effects.

In the LDT, participants were asked to decide, as quickly and as accurately as possible, if a string of visually-presented letters was an English word or not by pressing either the ‘I’ key on a computer keyboard (labelled Yes) or the ‘E’ key (labelled No). Stimuli remained on the screen until participants made a response. No feedback was provided. Stimuli were presented centrally on the screen using Arial font (12pt) in white colour against a grey background. Each stimulus was preceded by a central fixation cross presented for 250 ms. Participants started with eight practice trials to help them become familiar with the location of the response keys and trial format. In the experiment proper, there were 84 trials divided into 2 blocks. Each block of 42 trials included 2 warm-up trials and 40 experimental trials. These 40 trials consisted of 10 trials of each of the 4 stimulus types (i.e., regularly spelt words; irregularly spelt words; pseudo-homophone non-words; visually-confusable non-words) presented pseudo-randomly. That is, trial order was randomised, but with the constraint that no two items of the same stimulus type were presented consecutively. A break was provided between the two blocks.

Following completion of the four visual recognition tasks, participants filled in the Waterloo Handedness Questionnaire-Revised (Elias et al., 1998), and provided demographic data pertaining to whether they had an official dyslexia diagnosis, their age, gender and occupation. In total, it took approximately 45 min to complete all aspects of the study.

### Handedness

Scores on the WHQ-R ranged from − 53 to 72, with a mean of 43.22 (SD = 28.54), indicating a predominately right-handed sample.

## Results

All analyses described in this section were conducted using Jamovi (Version 2.3.13.0). Descriptive statistics overviewing general task performance are provided in Table 1 for the Lexical Decision Task (LDT) and Visual Recognition Tasks (VRTs). We first explored any general performance similarities and/or differences between tasks and stimulus types. We then tested hypotheses that if the recognition of words, faces, and other object categories share common neural underpinnings, then performance across tasks should be correlated. However, if these different forms of pattern recognition are distinct dissociable ability domains, then performance across tasks would not be hypothesised to be correlated. To identify potential commonalities in our behavioural data, two rounds of Spearman’s *ρ* correlations were computed: one for the response accuracy data and one for the response time data (for correct responses) from both the Lexical Decision Task (LDT) and Visual Recognition Tasks (VRTs). Subsequently, two principal component analyses were conducted on the same basis.

**Table 1:** Mean (Standard Deviation) accuracy and reaction time for correct trials in lexical decision and visual recognition tasks

		Accuracy (%)	Response time (ms)
Lexical decision tasks			
	Regularly-spelt words	95.45 (7.16)	938.27 (278.62)
	Irregularly-spelt words	92.45 (9.49)	922.56 (286.62)
	Pseudo-homophone non-words	94.45 (8.91)	1164.55 (494.71)
	Visually-confusable non-words	91.75 (12.74)	1171.59 (470.63)
Visual recognition tasks			
	Face	79.01 (15.86)	3461.80 (1024.96)
	Body	65.93 (11.35)	4308.98 (1455.05)
	Car	71.75 (17.07)	4932.80 (1502.43)

## Task performance similarities and differences

We used paired samples *t*-tests to explore similarities/differences in average task performance between tasks. In terms of response accuracy, participants were significantly more accurate in recognising regularly-spelt words compared to irregularly-spelt words, *t*(73) = 4.35, *p* < 0.001, *d* = 0.50, as well as pseudo-homophone non-words compared to visually-confusable non-words, *t*(73) = 3.24, *p* = 0.002, *d* = 0.37. Response accuracy differed significantly between all three VRT object categories, all *p*s < 0.001, and LDT accuracy was overall significantly higher than VRT accuracy, *t*(72) = 20.61, *p* < 0.001, *d* = 2.41.

For response times (analysed for correct responses only), however, participants were not significantly faster in recognising regularly-spelt words relative to irregularly-spelt words, *p* = 0.44, or pseudo-homophone non-words relative to visually-confusable non-words, *p* = 0.83. Thus, these data were collapsed together to form two superordinate ‘word’ and ‘non-word’ categories. Analyses of these data showed that participants correctly responded to real words significantly faster than to non-words, *t*(73) = − 6.05, *p* < 0.001, *d* = − 0.70. Additionally, mean response times were overall significantly faster for the LDTs compared to the VRTs, *t*(72) =  − 23.28, *p* < 0.001, *d* = − 2.72.

## Correlations

Results of the Spearman’s *ρ* correlations are presented in Table 2 for response accuracy and Table 3 for response times. For response accuracy, significant positive correlations were observed between face and car recognition (*p* = 0.01), car and body recognition (*p* = 0.01), body recognition and regularly-spelt words (*p* = 0.002) and faces and irregularly-spelt words (*p* = 0.04). Additionally, significant positive correlations were found between regularly- and irregularly-spelt words (*p* < 0.001), regularly-spelt words and pseudo-homophone non-words (*p* = 0.03), regularly-spelt words and visually-confusable non-words (*p* = 0.02), visually-confusable non-words and irregularly-spelt words (*p* = 0.010), and visually-confusable and pseudo-homophone non-words (*p* < 0.001).

**Table 2:** Spearman’s *ρ* for the response accuracy data

	Regular words	Irregular words	Pseudo-non-words	Confusable non-words	Faces	Bodies	Cars
Regular words	–						
Irregular words	0.68^***^ < *.001*	–					
Pseudo non-words	0.21^*^ ***0.03***	0.18 *0.05*	–				
Confusable non-words	0.22^*^ ***0.02***	0.24^*^ ***0.01***	0.68^***^ < *.001*	–			
Faces	0.09 *0.02*	0.20^*^ ***0.04***	0.02 *0.42*	− 0.06 *0.71*	–		
Bodies	0.36^**^ ***0.002***	0.17 *0.07*	0.10 *0.19*	0.04 *0.36*	0.11 *0.15*	–	
Cars	0.09 *0.21*	0.06 *0.29*	0.01 *0.44*	− 0.01 *0.54*	0.26^*^ ***0.01***	0.24^*^ ***0.01***	–

Significant correlations are highlighted in bold, and p values are presented in italics

*p < .05

**p < .01

***p < .001

**Table 3:** Spearman’s *ρ* for the response time data

	Regular words	Irregular words	Pseudo non-words	Confusable non-words	Faces	Bodies	Cars
Regular words	–						
Irregular words	0.82^***^ < *.001*	–					
Pseudo non-words	0.65^***^ < *.001*	0.70^***^ < *.001*	–				
Confusable non-words	0.59^***^ < *.001*	0.59^***^ < *.001*	0.86^***^ < *.001*	–			
Faces	0.29^**^ ***0.005***	0.18 *0.05*	0.29^**^ ***0.006***	0.31^**^ ***0.003***	–		
Bodies	0.24^*^ ***0.01***	0.12 *0.14*	0.21^*^ ***0.03***	0.27^**^ ***0.01***	0.71^***^ < *.001*	–	
Cars	0.16 *0.08*	0.06 *0.27*	0.16 *0.07*	0.17 *0.07*	0.74^***^ < *.001*	0.78^***^ < *.001*	-

Significant correlations are highlighted in bold, and p values are presented in italics

*p < .05

**p < .01

***p < .001

For response time, significant positive correlations were observed between face and body recognition (*p* < 0.001), face and car recognition (*p* < 0.001), body and car recognition (p < 0.001), face recognition and regularly-spelt words (*p* = 0.005), face recognition and pseudo-homophone non-words (p = 0.006), face recognition and visually-confusable non-words (*p* = 0.003), body recognition and regularly-spelt words (*p* = 0.010), body recognition and pseudo-homophone non-words (*p* = 0.030), and body recognition and visually-confusable non-words (*p* = 0.010). As with the accuracy data, RTs for regularly-spelt words were correlated with all further word types (i.e., irregularly-spelt words *p* < 0.001, pseudo-homophone non-words *p* < 0.001, visually-confusable non-words, *p* < 0.001, respectively). However, this pattern was repeated for all further word types, i.e., irregularly-spelt words and pseudo-homophone non-words (*p* < 0.001), irregularly-spelt and visually-confusable non-words (*p* < 0.001), and pseudo-homophone non-words and visually-confusable non-words (*p* < 0.001).

To sum, while several significant correlations were observed for both response accuracy and response times, the response time data showed a stronger pattern of within domain correlations (e.g., words, objects) and cross-domain correlations, particularly for faces, bodies, regularly-spelt words and both non-word categories.

## Principal component analyses

To explore further and potentially allow for a clearer understanding of commonalities, two principal component analyses (PCA) were conducted (with Oblimin rotation) on the response accuracy and response time data. These analyses were conducted to reduce dimensionality (i.e., reduce the number of variables involved) to allow identification of potential latent constructs that might account for the variance in those variables involved.

For the response accuracy data, the Kaiser–Meyer–Olkin (KMO) test showed higher sampling adequacy for the VRT (Faces, 0.65; Bodies, 0.64; Cars, 0.62) than for the LDT (Regularly-spelt words, 0.55; Irregularly-spelt words, 0.55; Pseudo-homophone non-words, 0.50; Visually-confusable non-words, 0.49).^1^ Nevertheless, in the present analyses, two components were identified with eigenvalues greater than 1.5 (adopting > 1 as the acceptable standard). These two components accounted for 55.2% of the variance in accuracy (Component 1, 29.5%; Component 2, 25.7%). Component loadings for the different variables are shown in Table 4. Component 1 appears to reflect ‘known categories of word/object recognition’, whereas component 2 appears to map onto ‘novel (word) stimuli’. The inter-component correlation was non-significant and reflected a very small correlation between the two components (0.11). This result suggests that functional overlap in known-object recognition may not necessarily extend to novel object/stimulus categories.

**Table 4:** Component loadings for the Lexical Decision and Visual Recognition Task response accuracy data

Component			
	1	2	Uniqueness
Regular words (% correct)	0.83		0.30
Irregular words (% correct)	0.82		0.31
Pseudo non-words (% correct)		0.93	0.11
Confusable non-words (% correct)		0.95	0.09
Faces (% correct)	0.51		0.73
Bodies (% correct)	0.52		0.73
Cars (% correct)	0.40		0.83

For the response time data, sampling adequacy was higher for both the VRT (Faces, 0.77; Bodies, 0.73; Cars, 0.62) and the LDT data (Regularly-spelt words, 0.75; Irregularly-spelt words, 0.73; Pseudo-homophone non-words, 0.70; Visually-confusable non-words, 0.68). This indicates that the response time variables clustered more strongly into factors than the response accuracy variables. It further is consistent with the two components observed, including the higher loadings onto the two observed components (Table 5). To expand, the PCA revealed two components with eigenvalues greater than 2, which accounted for 44.9% and 36.8% of the variance in reaction time, respectively. Component 1 reflects visual word recognition (the LDT task), whereas Component 2 reflects object recognition (the VRTs). The inter-component correlation was 0.23, again demonstrating a somewhat small correlation between the two components. In other words, the degree of domain generality in visual object recognition (i.e., the VRT response times) is related, albeit not strongly, to visual word form recognition (i.e., the LDT response times).

**Table 5:** Component loadings for the lexical decision and visual recognition task response time data

Component			
	1	2	Uniqueness
Regular words (RT)	0.86		0.22
Irregular words (RT)	0.90		0.21
Pseudo non-words (RT)	0.90		0.16
Confusable non-words (RT)	0.86		0.23
Faces (RT)		0.89	0.17
Bodies (RT)		0.90	0.16
Cars (RT)		0.95	0.10

**Fig. 2:**
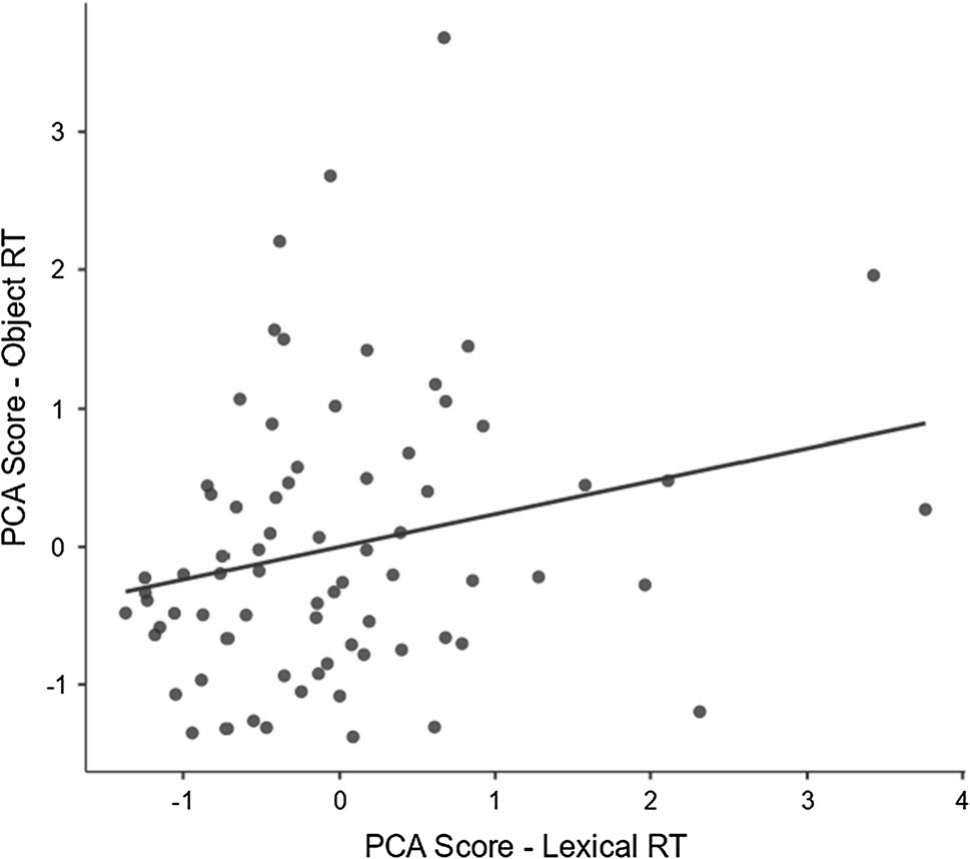
Scatterplot of the correlation between PCA component scores resulting from the PCA analysis of the response time data (Table 5)

To confirm this, PCA component scores were extracted for each participant from both analyses and entered into a round of Spearman’s *ρ* correlations. As reported above, the results produced no significant correlation for response accuracy components (Words, Non-Words and Objects), but a significant correlation between the lexical (i.e., LDT) response time component and the object (i.e., VRTs) response time component, *r* = 0.23, *p* = 0.02 as illustrated in Fig. 2. This confirms that a small but significant correlation exists between how similar response times are for the four LDT visual word recognition tasks, and how similar they are between recognition for non-lexical object categories (i.e., faces, bodies and cars).

## Discussion

Previous small-sample behavioural and neuroimaging research has proven equivocal as to whether different forms of pattern recognition form distinct dissociable ability domains or share common neural underpinnings. Thus, the purpose of the present research was to explore relationships between body, face, car and (four types of) word recognition, through investigation of both accuracy and reaction time metrics. Whilst we observed significant correlations across domains for both recognition accuracy and response time, providing some support for at least partial domain-general pattern recognition, correlations differed in size and significance as a function of performance measure (i.e., response accuracy or response time). To expand, we found many more significant correlations across categories for the reaction time data than for the accuracy data. Therefore, to explore further, and potentially identify commonalities between our many task variables, we conducted PCA analyses for each performance measure. These analyses revealed a two-component model for the accuracy data and a two-component model for the reaction time data. However, how the various word and object recognition tasks fitted these factors varied considerably. These results are discussed below, considering first the correlations and then the PCA analyses for each performance measure.

In terms of response accuracy, a small but significant positive correlation was found between regularly-spelt word recognition and body recognition accuracy, and between irregularly-spelt word recognition and face recognition accuracy. This suggests a potential functional overlap between these abilities. Further small, positive, significant correlations were also found between car and face recognition, and between car and body recognition, but correlations between word categories were more mixed (e.g., a strong correlation between regularly spelt and irregularly spelt words, but no correlation between irregularly-spelt words and pseudo-homophone non-words). The PCA response accuracy data complemented these findings by identifying a component which appeared to reflect ‘known’ categories of word/object recognition (and included regularly- and irregularly-spelt words, as well as faces, bodies and cars) and a component that reflected ‘novel’ (word) recognition (comprised of only the pseudo-homophone non-words and confusable non-words).

In general, these results appear to suggest that while functional overlap may exist between different object and word recognition domains, consistent with some research (e.g., Asperud et al., 2019; Dundas et al., 2013; Sigurdardottir et al., 2015), the patterns of overlap are likely complex. Indeed, the results here seem to suggest a role for expertise (Gauthier et al., 1999). To expand, as we found ‘known stimuli’ clustered together regardless of whether they represented words or objects, this could be taken as evidence of a shared ‘pattern’ recognition mechanism when expertise is achieved. This would be supported by the finding that visually-confusable non-words did not form part of this component. Indeed, whilst visually-confusable non-words are visually similar to real words, one would not recognise them immediately on inspection (e.g., weed vs. weeb), consistent with established lexicality effects (e.g., Balota and Spieler 1999). This finding is further supported by the fact that only pseudo-homophone non-words and visually-confusable non-words formed the second component, as both types of stimuli were unknown and novel. Importantly, in lexical decision tasks participants need to decide if visually-presented letter strings are known words or made-up letter strings (i.e., non-words). Thus, the lexical decision task allows investigation of detailed visual processing required for reading, including a participant’s visual word recognition ability. This task potentially constitutes a functional bridge linking word recognition to other types of visual processing—such as faces and/or other object categories—given real words should be instantly recognizable patterns. In the present study, there was a clear difference in component structure depending upon whether words were known (i.e., real regularly and real irregularly spelt words) or unknown (i.e., pseudo-homophone and visually-confusable non-words). Importantly, the known words clustered with other known stimuli (faces, cars, body parts), as opposed to comprising a further third PCA component. This finding hence supports a domain-general explanation of pattern-recognition as the known stimuli, regardless of whether words or objects, clustered together.

In addition, the correlation between the “known object” and the “novel object” components resulting from the PCA on the response accuracy data was small and not significant, potentially suggesting that domain-generality does increase with *familiarity* with object categories. That is, as we become more familiar with object categories, we might develop processes for their recognition that depend on common features they might share with objects in other familiar categories. Thus, it can be argued that our accuracy findings support recognition shaped by learning and experience (e.g., Aguirre et al., 1998; Gauthier et al., 2014), rather than category recognition as innate and functionally specific (e.g., Hannagan et al., 2015; Kamps et al., 2016).

Considering now the response time data (analysed only for correct responses), we found many more significant correlations across our different categories of word and object recognition tasks. For example, face recognition response times were correlated with regularly-spelt words, pseudo-homophone and visually-confusable non-word response times, as were body recognition times. However, the strongest correlations were observed ‘within’ categories. To expand, performance was strongly and significantly correlated across the four word tasks (i.e., lexical decision task components), as was performance across the three object recognition tasks. These findings therefore hint at functional specificity; a finding that was confirmed by our PCA analyses. That is, the response time PCA results neatly divided the words (all LDT categories) and objects (all VRT categories) into two distinct components. In contrast to our accuracy measure, these findings suggest a role for functional specificity (Almeida et al., 2020; Kanwisher et al., 1997) and the idea that, potentially, there are brain regions specialised for at least word recognition (e.g., Dehaene and Cohen 2011) and object recognition (e.g., Epstein et al., 1999; Hannagan et al., 2015; Kamps et al., 2016).

At first glance, the accuracy and response time results appear quite contradictory. However, when one considers that for response time data we only analysed correct responses (as is the norm), it can be assumed that in this situation we are tapping ‘known’ stimuli or ‘known’ processes. Thus, in this respect, as with accuracy, results could reflect expertise. Therefore, one explanation for our accuracy results is that expertise allows for functional specificity to develop and the clustering of ‘word patterns’ together and, accordingly, the clustering of ‘object patterns’ together. This is actually quite a common finding in pattern recognition research more broadly. For example, functional specificity has not only been found in the case of words (Cohen et al., 2000) and faces (e.g., Kanwisher et al., 1997; Sergent et al., 1992), as cited previously, but also in the case of other forms of pattern expertise. For instance, the FFA is activated by chess pieces in chess experts (Bilalic et al., 2011), and by cars and birds in car and bird experts, respectively (Gauthier et al., 2000a, b). Added to this, it must be noted that for response time analyses, we observed that for each participant, the two PCA components (i.e., word and object recognition) were significantly correlated. This might suggest that, to an extent, whether pattern recognition is domain-specific or domain-general is a result of individual differences. If this is the case, then again it could be argued that expertise is one of the underlying factors that shape neural development and henceforth regional functional specificity.

This stated, it should further be noted that correct responses to lexical items (i.e., words and non-words) were executed significantly faster than correct responses to non-lexical items (i.e. faces, bodies and cars). So, differences in average response times between tasks and between object categories (and why these tasks clustered together in the PCA analyses) might, at least partly, reflect differences in task demands rather than domain specificity. In other words, the visual complexity of a face or car compared to a string of letters would influence the time required to process a stimulus, even if processed in a domain-general way.

To sum, whilst we set out to investigate if the recognition of words, faces and/or other object categories share common neural underpinnings, or if these different forms of pattern recognition represent distinct dissociable ability domains, the current results provided a complex picture of domain interactions between words, faces and other object categories. For the most part, reaction times and accuracy correlated across tasks, which is partly consistent with proposals for domain-general object recognition mechanisms (e.g., Behrmann & Plaut 2013; Gauthier et al., 2014). This finding was further consistent with the PCA response accuracy data that demonstrated two recognition components/factors, not distinguished by super-ordinate task type (i.e., by words or objects), but by known versus unknown ‘patterns’. As such these findings lend some behavioural support to neuroscientific findings challenging domain-specificity of the FFA (e.g., Bilalić et al., 2016) or VWFA (e.g., Vogel et al., 2014) and instead suggest a role for expertise (e.g., Gauthier et al., 1999; 2014; Martens et al., 2018). Conversely, and seemingly consistent with functional specificity (e.g., Kamps et al., 2016; Kanwisher et al., 1997), PCA response time results did neatly reflect a division between word recognition and object recognition. However, these findings could reflect expertise given that the response time data reflected ‘correct’ and arguably known stimuli/processes and/or simply task complexity. Thus, taking all findings together, our findings reflect pattern recognition as a complex process involving both elements of domain-general and domain-specific processing. We tentatively theorise that expertise is a key shaping factor bridging the two, with expertise (and individual differences) shaping how brain regions respond selectively, and more efficiently, not only to a specific category of object but to multiple categories whose members share (or can meaningfully be decomposed into) similar features such as visual primitives.

## Limitations and future directions

Our research is not without limitation. For example, while sample size was determined from existing correlational evidence (Pinel et al., 2015), it is possible that it may have been inadequate to reliably estimate component structures via PCA. Future work should therefore aim to: (i) replicate these findings in larger samples; and (ii) ensure tasks are matched on difficulty/complexity. This would allow for more robust exploration of the possibility that the degree of domain-generality (or domain-specificity) in behavioural performances might, to an extent, be a function of individual differences. Certainly, in any future study, data on familiarity with specific object categories, as well as languages spoken, age, etc., may also prove insightful to include in analyses.

Related to this, studies with individuals with dyslexia, including measures of an individual’s reading proficiency, are likely to further expand our understanding of the functional similarities/differences in visual processing across a range of visual pattern categories. Indeed, if pattern recognition reflects domain-general processing emerging from visual similarities (or domain-specific processes emerging from domain-general processing), for novel stimuli we might expect to see performance differences between dyslexic and non-dyslexic individuals, with the former group performing more poorly. Whilst in the present study we did attempt to recruit dyslexic individuals, numbers were too low to allow meaningful analyses. Thus, future research specifically recruiting dyslexic individuals could provide meaningful theory understanding and advancement.

Finally, neuroimaging methods with high temporal resolution (such as magnetoencephalography) could be used to explore the time-course of visual processing of words, faces and other object patterns. Specifically, if domain-general processing emerges from visual similarities (or domain-specific processing emerges from domain-general processing), then one would expect to see more general brain activation on first presentation of a visual pattern (e.g., perhaps a variety of bilateral visual brain regions activated), but then specific regional activation when that pattern of visual features, or similar ones, is recognized, or with increased familiarity, (e.g., a specific hierarchical and lateralized visual brain region). The discussion of the new evidence produced by the current study has, therefore, opened up intriguing new avenues for future research into the nature of visual object recognition across diverse domains.

## Data Availability

Raw data will be made available upon request. None of the experiments was pre-registered.

## Footnotes

1 Sampling adequacy is a measure of the strength of the partial correlations between pairs of variables after controlling for all other variables. Values above 0.6 are generally considered acceptable (Hair et al., 2009). Additionally, although low KMO values are less problematic for PCA than for FA, they could still indicate that a larger sample size is needed to reliably estimate the factor structure.

## Code availability

N/A.
